# Supplementary material for: Characterization of Systemic and Culprit-Coronary Artery miR-483-5p Expression in Chronic CAD and Acute Myocardial Infarction Male Patients
Source: Int J Mol Sci. 2023 May 10;24(10):8551. doi: 10.3390/ijms24108551 (PMC10218121; doi:10.3390/ijms24108551)
Supplement: Supplementary file 1 [file ijms-24-08551-s001.zip › ijms-2264561-supplementary.pdf]

Supplementary Materials:

Supplementary Table S1: Differentially expressed miRNAs detected by NGS analysis in sera from STEMI patients *versus* patent controls. Fold change and log2 fold change values, as well as *p*-values are shown for each of the 15 miRNAs. NGS: Next Generation Sequencing; RPM: reads per million; STEMI ST-elevation myocardial infarction.

miRNA	mean expression (RPM)	log2 fold change	Fold Change	*p* Value
**hsa-miR-1-3p**	285.58	1.78	3.44	0.0023
**hsa-miR-150-5p**	14736.14	1.19	2.28	0.0031
**hsa-miR-483-5p**	1259.12	-0.97	0.51	0.0041
**hsa-miR-1229-5p**	92.82	3.51	11.38	0.0064
**hsa-miR-4723-5p**	125.51	5.57	47.59	0.0068
**hsa-miR-1299**	29.87	2.15	4.43	0.0101
**hsa-miR-3168**	530.26	1.16	2.23	0.0109
**hsa-miR-2467-5p**	1040.32	-1.03	0.49	0.0146
**hsa-miR-122-5p**	27183.72	-0.69	0.62	0.0232
**hsa-miR-361-3p**	1507.20	0.76	1.69	0.0274
**hsa-miR-29a-3p**	4190.70	0.70	1.63	0.0278
**hsa-miR-29b-3p**	237.73	1.06	2.09	0.0347
**hsa-miR-643**	82.72	2.85	7.2	0.0411
**hsa-miR-4786-3p**	64.04	4.17	17.98	0.0469
**hsa-miR-142-5p**	5919.69	0.70	1.63	0.0473
